# Effects of Visual Priming on Taste-Odor Interaction

**DOI:** 10.1371/journal.pone.0023857

**Published:** 2011-09-28

**Authors:** Marije van Beilen, Harold Bult, Remco Renken, Markus Stieger, Stefan Thumfart, Frans Cornelissen, Valesca Kooijman

**Affiliations:** 1 Top Institute Food and Nutrition, Wageningen, The Netherlands; 2 Center for Innovative Consumer Studies, Ede, The Netherlands; 3 Laboratory for Experimental Ophthalmology, University Medical Center, University of Groningen, Groningen, The Netherlands; 4 School of Behavioral and Cognitive Neurosciences- NeuroImaging Center, University Medical Center, University of Groningen, Groningen, The Netherlands; 5 Profactor GmbH, Steyr-Gleink, Austria; 6 Department of Neurology, University Medical Center, University of Groningen, Groningen, The Netherlands; 7 Food and Biobased Research, Wageningen University and Research Centre, Wageningen, The Netherlands; 8 Division of Human Nutrition, Wageningen University and Research Centre, Wageningen, The Netherlands; Alexander Flemming Biomedical Sciences Research Center, Greece

## Abstract

Little is known about the influence of visual characteristics other than colour on flavor perception, and the complex interactions between more than two sensory modalities. This study focused on the effects of recognizability of visual (texture) information on flavor perception of odorized sweet beverages.

Participants rated the perceived sweetness of odorized sucrose solutions in the presence or absence of either a congruent or incongruent visual context. Odors were qualitatively reminiscent of sweet foods (strawberry and caramel) or not (savoury). Visual context was either an image of the same sweet foods (figurative context) or a visual texture derived from this product (non-figurative context). Textures were created using a texture synthesis method that preserved perceived food qualities while removing object information. Odor-taste combinations were rated sweeter within a figurative than a non-figurative context. This behaviour was exhibited for all odor-taste combinations, even in trials without images, indicating sustained priming by figurative visual context. A non-figurative context showed a transient sweetening effect. Sweetness was generally enhanced most by the strawberry odor.

We conclude that the degree of recognizability of visual information (figurative versus non-figurative), influences flavor perception differently. Our results suggest that this visual context priming is mediated by separate sustained and transient processes that are differently evoked by figurative and non-figurative visual contexts. These components operate independent of the congruency of the image-odor-taste combinations.

## Introduction

Investigation of the effects of visual context on flavor perception has been limited to the influence of colour, in bimodal paradigms. If we present a three-modal sensory integration paradigm including both figurative and non-figurative images, we can investigate the role of other visual characteristics such as visual texture and memory (recognizability of the product) on flavor in multimodal sensory integration processes.

Multi-modal integration is the perceptual consolidation of activations of different sensory modalities. Just before and during food intake, visual, gustatory, olfactory, tactile and auditory information are processed by the individual, resulting not only in unimodal visual, olfactory and haptic perceptions but also in an integrated percept of the food product. Schifferstein showed that multiple modalities are participating in different ways in the experience of physical objects - and food products in particular. Although in real-life product evaluation, vision and haptic perception appear to be the dominant modalities [1], the immediate sensory impression of foods is dominated by taste, smell and oral haptic perception. In the perception of sensory properties of foods, these latter modalities are known to interact: odorants may enhance or suppress taste [2] [3] and vice versa [4], food texture affects taste [5] [6] and food texture affects smell [7], [8] and vice versa [8].

An example of the contribution of vision to the multi-modal perception of a food product is the effect of colour on flavor perception [9] [10], with flavor being the unified impression of odor and taste. More intense colouring results in the perception of more intense flavor [11], [12] [13] whereas incongruent pairs of colour and flavor (e.g. a red colour and a banana flavor) hamper flavor identification [13]. The exact nature of effects of colour on flavor perception is still not completely resolved [14], [15] [16]. One possible explanation for this inconsistency is that the occurrence of colour-flavor interactions may rely heavily on the specificity of the stimulus associations adopted by the participants involved [17] [18]. For example, Dubose et. al. [13] showed colour-induced flavor enhancement for orange-flavored drinks but not for cherry-flavored drinks. Hence, colour-flavor congruency is a factor in flavor perception but its influence is not easily predicted without knowledge of a participant's experience with the used colour-flavor combination [10].

The same holds for the effects of colour on the perception of odors in the absence of concurrent taste stimulation: stronger cross-modal effects are observed for (semantically) associated colour-odor pairs compared to un-associated pairs [19] [20]. Neuro-physiological studies of sensory integration processes of congruent information show that relevant brain activity elicited by one sensory modality is potentiated by the concurrent stimulation of the other modality. Also, ratings are faster and more accurate when odor stimuli are paired with semantically congruent visual cues (i.e., ice-cream for vanilla odor) compared to semantically incongruent visual cues [21] [22]. In fact, colour may even influence odor perception to the extent that an incongruent colour (white wines coloured red) dominates the odor evaluation, leading to misinterpretations of odors (causing white wine as be judged to be red wine, even by wine experts) [23].

In spite of all scientific attention to colour in the study of cross-modal interactions, real life vision entails more than simply processing the colour of a product. In fact, other features such as movement, shape, or texture also matter for the perception of concurrent olfactory stimuli [24] [25]. Also, the sensory information is combined with existing knowledge (memory) [21]). Therefore, in this view, sensory integration includes memory processes, in terms of the recognizability of food-products and congruency between different modalities. In this study we aimed to investigate effects not just of colour but of a well-defined complex visual context on the perceived flavor of odorized drinks. While colours and odors influence flavor perception each in their own right, priming observers with images of sweet food products may affect the cross-modal influence of odors on the flavor of food products even more. To test this explicitly, the amount of visual semantic information was manipulated by showing participants images of actual sweet food products food (figurative condition), or images without recognisable food products but that contained the visual texture, hue and colours of the sweet food products (non-figurative condition). The recognizability of food products is also manipulated by varying the congruency levels of stimuli. For this purpose, we used congruent odors (e.g. strawberry image with strawberry odor), situational incongruent odors (e.g. strawberry image with caramel odor) and definitely incongruent odors (e.g. strawberry image with savoury odor) to investigate the effects of images on sweetness of odorized sucrose solutions.

We tested the following hypotheses:

1. Odors of sweet products enhance the sweetness of sucrose solutions.
2. Priming participants with images of sweet products can further enhance this sweetness perception.
3. Taste enhancement through visual priming depends on semantics: figurative images will enhance sweetness ratings more than non-figurative images.
4. Visual priming enhances sweetness ratings most when the concurrent odor is congruent with sweet taste and with the image, less when the odor is congruent with sweet taste and not congruent with the image and least when the odor is neither congruent with sweet taste, nor with the odor.

## Materials and Methods

### Ethical Statement

This study was conducted according to the principles expressed in the declaration of Helsinki and was approved by the Medical Ethical Committee of the University Medical Centre Groningen. All participants gave written informed consent.

### Participants

Forty-eight healthy participants (mean age 23.4, SD 4.8, range 19–54 years, 20 male) were randomly assigned to two groups of 24 participants. All participants reported that they had never experienced problems regarding olfactory functioning and reported to have normal visual, taste and odor perception according to their own judgement. Colour blinds, smokers and participants with a nose cold were excluded from participation. All participants completed the experiment.

### Taste-odor stimuli

Odorants used were a commercially available strawberry odor (QL83777; Quest International, The Netherlands), 4-hydroxy-2,5-dimethyl-3(2H)-furanone (furaneol, Sigma-Aldrich, The Netherlands), which has a retronasal odor detection threshold 50·10^−9^ (w/w) in water, and 5-ethyl-3-hydroxy-4-methyl-2(5H)-furanone (Abhexon, Sigma-Aldrich, The Netherlands), which has a retronasal odor threshold of 3·10^−9^ w/w in water. In the oral stimuli, furaneol is diluted to 2.0·10^−6^ w/w, the strawberry odor to 100·10^−6^ w/w and Abhexon to 0.20·10^−6^ w/w. At these concentrations, the odor quality of furaneol can be described as “Caramel-like”. Strawberry odor, at the used concentration can be described as “Strawberry” and Abhexon as “Savory/Bouillon”. In the present study, these odors will be referred to as such. Ratings from a well-trained sensory-analytical panel consisting of eleven members revealed no subjective differences between the three odors at the used solution concentrations. Combined taste-odor stimuli were six aqueous solutions of 4% w/w sucrose (crystalline, purchased at a local retailer), 6% w/w sucrose, 8% w/w sucrose, 6% w/w sucrose with “Strawberry odor”, 6% w/w sucrose with “Caramel odor” and 6% w/w sucrose with “Savoury odor”, respectively.

### Visual stimuli

**Figure 1:**
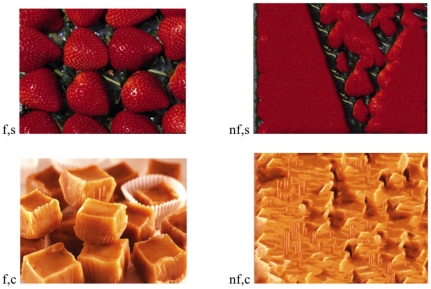
Figurative and Non-Figurative (nf) images of Strawberry (s) and Caramel (c).

Visual stimuli consisted of an image with strawberries and an image depicting caramel (figure 1). These images were selected as follows: a separate evaluation experiment, 80 participants (students at the University of Groningen) rated these images as being among the two most ‘congruent-with-strawberry-odor’ and the two most ‘congruent-with caramel-odor’ respectively, out of a total of 145 images depicting sweet foods or beverages with various colours. These four images were presented to 20 participants while they simultaneously tasted an 8% (w/w) sucrose solution in water. The two images that enhanced the perceived sweetness most were selected for this study. In the main visuo-olfactory-gustatory interaction study, the images were presented both in upright and 180° rotated versions.

From these two figurative images, two non-figurative images were constructed by applying sample-based texture synthesis to remove object information. Sample-based texture synthesis involves the randomization of large-scale figural dependencies, while preserving small-scale neighbourhoods. By doing so, the large-scale object information is destroyed, while image macro-properties, such as colour and surface statistics remain stable (Figure 1). With sample-based texture synthesis, an original input sample is used to synthesize a new texture, which looks like being generated from the same underlying statistical process as the input sample. We used a recent approximate nearest neighbour search method [26] to speed up neighbourhood search, enabling us to synthesize high quality images in reasonable time. The distance between multi-resolution neighbourhoods is computed by the summed squared distance between all neighbourhood pixels [27]. Furthermore, by varying parameters such as neighbourhood size and weighting, we fine-tuned the “degree” of figurativeness. Accordingly, we synthesized non-figurative images that did not contain recognizable objects but which preserved their textural food qualities. Importantly, our method does not introduce new sharp edges (as do more conventional block scrambling methods, as in e.g. [28].

### Oral stimulus presentation

To achieve the required high level of stimulus control, a fully automated presentation system was used, based on a single computing unit controlling the presentation of the oral stimuli, the screen instructions, the visual stimuli and the warning beeps for the panellists [8]. Liquids were pumped by an array of eight identical membrane-liquid pumps (KNF Stepdos FEM03.18RC, KNF Verder, Vleuten, The Netherlands) through 10-m Teflon tubing (1.6 mm inner diameter) and mixed in an 8 to 1 channel manifold. Each feeding tube was provided with an inline check valve immediately before the manifold to prevent cross-contamination of feeding tubes. From the manifold, a 5-cm tubing (0.8 mm inner diameter) with a 26-µL dead volume conducted liquid stimuli to the tip of the tongue of the participant. Each of the liquid stimuli and the rinsing water were presented at a flow rate of 20 mL/min. Consequently, the presentation of 1.0-mL stimuli took 3 seconds and the presentation of 1.33 mL aliquots of rinsing water took 4 seconds. Since stimulus presentations were always preceded by rinsing water in the previous trial the dead volume of the mouthpiece caused that all stimuli were diluted with 2.5% water. We will further refer to this apparatus as a “gustometer”.

### Procedure

Participants were seated in an up-right position in a comfortable chair. The outlet of the gustometer was mounted on a tripod and manoeuvred in front of the mouth of the participant. Instructions and images were presented against a grey background on a 20 inch flat-panel LCD screen (36×29 cm) placed 100 cm in front of the participant. Images were centred on the screen, and occupied approximately 11 by 9 degrees of visual angle. The room was blinded and only illuminated by the stimulus display.

**Figure 2:**
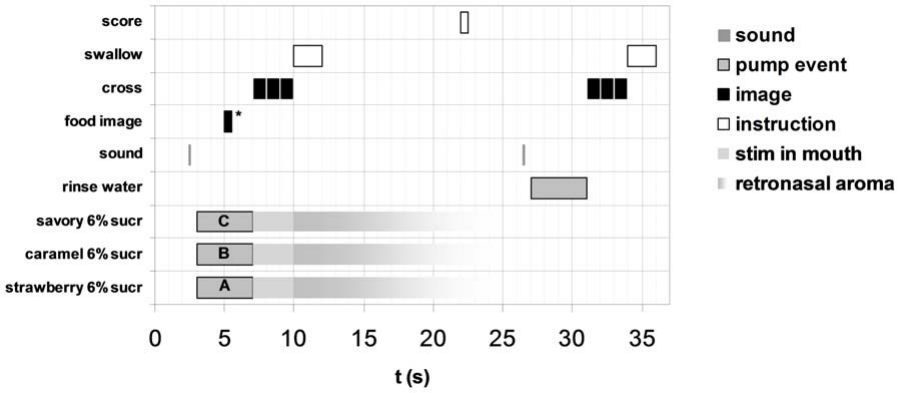
Timed events for each experimental trial. Six % sucrose solutions had equal probabilities of containing Strawberry (A), Caramel (B) or Savory (C) Odors. Durations for stimulus presence in the mouth and retronasal odor presence are idealized on basis of expectations in case instructions are followed correctly. Food images were a selection of Figurative Strawberry, Non-Figurative Strawberry, Figurative Caramel and Non-Figurative Caramel images.

Experimental trials consisted of a series of acoustic, visual, and gustatory events, as shown in Figure 2. First, participants were familiarized with the experimental procedure using three arbitrary non-food pictures instead of the figurative and non-figurative food images. Then, different aqueous sucrose solutions (4%, 6%, 8%) were presented (twice each) to indicate the range of sweetness intensities that participants could expect in the study (i.e. 6 calibration trials). Subjects were instructed to rate sweetness intensities verbally by magnitude estimation, applying a range from 0 (least sweet imaginable) to 100 (most sweet imaginable). Subjects then rated the sweetness intensities of the same three odorized sucrose solutions (twice each), in a randomized order (total 6 no-image trials). After a short break, subjects evaluated combinations of food-images with odorized sucrose solutions in randomized order. The images (2) were shown twice in combination with each odor (total: 12 experimental trials). See also Table 1 for an overview of experimental conditions. During the presentation of the oral stimuli, the image was shown for 4 seconds. After this, a fixation cross was shown in the centre of the screen. Next, participants received the instruction to swallow, which had to be initiated as soon as possible. Immediately after swallowing, participants rated the overall stimulus sweetness, which were noted by the experiment leader. Care was taken not to use any terminology suggesting that different stimuli (i.e. separate odors and flavors) were to be rated. In between stimuli, water was used for mouth rinsing.

**Table 1:** Design including Odor and Image conditions for two groups (Figurativeness).

Odor			strawberry	caramel	savory
**Image**					
	**figurative (n = 24)**	**strawberry**	c	si	di
		**caramel**	si	c	di
		**no-image**	baseline	baseline	baseline
	**non-figurative (n = 24)**	**strawberry**	c	si	di
		**caramel**	si	c	di
		**no-image**	baseline	baseline	baseline

c: congruent; si: situational incongruent; di: definitely incongruent.

To prevent non-figurative images from being interpreted as figurative images by panellists after being exposed to the figurative versions of the images, the figurative and non-figurative images were presented to the two different panels of 24 observers.

Participants were required to answer the following standardized questions afterwards: 1. Did you notice an odor during the experiment? 2. Do you think that the flavors matched with the images? 3. Did you recognize what the picture showed?

### Design

The main study consisted of a 3×3×2 design with the within-participant factors Image (Strawberry, Caramel, No Image: fixation cross/baseline) and Odor (Strawberry, Caramel and Savoury) and between participant factor Figurativeness (Figurative image, Non-Figurative image). All within-participant factors were tested in a full-factorial randomized design. The order of the presentation of stimuli with variable levels of within participant factors was randomized for each participant. The between participant factor Figurativeness varied between groups of participants (n = 24 per group). Note that although this design aims at testing main effects of Odor, Image, Figurativeness and the interactions between these factors, tests of the level of congruency between images and odors can be prepared for the same design by defining congruency categories for all odor-image combinations (see Table 1).

### Data-analysis

A Kolmogorov-Smirnov test was used to test for deviations of normality for each variable and within each participant.

To check for rating biases in the sweetness ratings of the two groups of participants (i.e. groups of subjects participating in the Figurative- and the Non-Figurative conditions), we subjected the sweetness ratings of the initial sucrose-only stimuli to a 2-way repeated measures ANOVA with within-participant factor Sucrose level (4%, 6% and 8% (w/w)) and between-participant factor Figurativeness (figurative images, non-figurative images). This analysis was performed on the raw data obtained in the sucrose only conditions. Note that Figurativeness here indicates group membership only since no images have been shown yet to participants at this stage.

Next, to correct for scaling differences between participants, the data were normalized. Normalized ratings for each participant at trial i (R_i,nor_) were calculated: R_i.nor_ = ((R_i_−R_min_)/(R_max_−R_min_)) * 100), with R_i, nor_ being the normalized rating for trial i, R_i_ the raw score for trial i, R_max_ the maximum rating for the individual participant over all trials, and R_min_ the minimum rating for the individual participant over all trials (from here on referred to as ‘normalized ratings’).

Subsequently, sweetness ratings for each participant were referenced to the 6% sucrose-only baseline condition by subtracting the average 6% sucrose-only ratings from the ratings obtained in the subsequent experiment involving Image and Odor manipulations (from here on referred to as ‘re-referenced’ ratings).

The resulting normalized ratings were subjected to a full-factorial 3-way repeated-measures ANOVA testing effects of the within-participant factors Image (None, Strawberry, Caramel) and Odor (Strawberry, Caramel, Savoury) and the between-participants factor Figurativeness (Figurative image, Non-Figurative image). Post-hoc comparisons were made to evaluate the influence of Odor and figurative Image context (rather than the nature of the in-trial visual priming) on taste by testing whether the re-referenced sweetness ratings in the non-image conditions deviated from zero for either of the 3 Odor levels and the two Figurativeness (context) groups. This comparison was made by repeated-measures ANOVA on re-referenced sweetness ratings of sessions in which no images were shown with Odor as a within-participant factor and Figurativeness as between-participant factor. Additional post hoc comparisons examined the effects of images on sweetness ratings of image conditions (Strawberry, Caramel relative to No Image conditions), over Odor and Figurativeness levels. P-values were Bonferroni-corrected for multiple comparisons. To visualize these effects of image presentation relative to the no-image condition, averaged sweetness ratings obtained in the Odor+No Image conditions were subtracted from averaged ratings obtained in the corresponding Odor+Image (Strawberry or Caramel) conditions. This subtraction was performed for all Odor conditions (Strawberry, Caramel, and Savoury).

Finally, effects of odor-image congruency were performed by repeated-measures ANOVA, including the within-participant factors Congruency (Congruent, Situational Incongruent, Definitely Incongruent) and Image (Strawberry, Caramel) and the between-participants factor Figurativeness.

## Results

The Kolmogorov-Smirnov test indicated that the distribution of the sweetness ratings did not deviate from normality within each variable and each participant.

### Questionnaires

Results of a short questionnaire presented after the tasting sessions revealed that 79% of the participants reportedly did not notice any odor. Due to the small size of the group that did notice an odor, it was not possible to compare both groups on differences in experimental trials or subsequent answers on the questionnaire. Furthermore, the estimated degree of congruency between images and the sweet taste was rated by 13% as totally absent, by 15% as always present and by 65% as present in some trials but absent in others. Of the non-figurative group, 25% of the participants did not recognize the semantic value of the image, 25% did, and 50% recognized it partly.

Because a paired sample t-test showed no significant differences between sweetness ratings for upright and rotated images, results were averaged over image rotation categories.

### Effects of sucrose concentrations and Figurativeness groups on sweetness

Repeated measures ANOVA of sweetness ratings for the sucrose only stimuli (4%, 6% and 8% sucrose) indicated no differences between Figurativeness groups [F(1,46) = 0.005; p = 0.94]. Note that these trials contained no odors and were presented prior to image-present trials. In line with expectations, sucrose concentration affected sweetness ratings significantly (F(2,92) = 87.2; p<0.001) by enhancing sweetness ratings for increasing sucrose concentrations (Table 2).

**Table 2:** Sweetness rating (0–100).

		All (48*)	Figurative (24*)	Non-figurative (24*)
		Mean (sd)	Mean (sd)	Mean (sd)
	Sucrose 4%	29 (16)	31 (15)	28 (18)
	Sucrose 6%	42 (20)	41 (18)	43 (22)
	Sucrose 8%	52 (20)	51 (19)	53 (20)
Baselines	Strawberry		58 (20)	51 (21)
	Caramel		56 (16)	46 (21)
	Savory		55 (20)	46 (22)
Conditions				
C	s_s**		57 (21)	55 (20)
	c_c		56 (21)	51 (23)
SI	s_c***		55 (20)	50 (23)
	c_s		56 (19)	52 (22)
DI	s_sa		54 (21)	52 (23)
	c-sa		56 (22)	51 (23)

*number of subjects;

**C = congruent, SI = situational incongruent, DI = definitely incongruent;

***for example: S_C: strawberry image+caramel odor.

### Effects of Image, Odor and Figurativeness on sweetness

Repeated measures ANOVA on normalized and re-referenced sweetness ratings for stimuli presented in image-present contexts revealed main effects (see also Table 3) of Image [F(2,92) = 3.2; p = 0.045], Odor [F(2,92) = 5.4; p = 0.006] and Figurativeness group [F(1,46) = 5.5; p = 0.023]. In addition, significant interactions were observed for Figurativeness×Image [F (2,92) = 5.0; p = 0.009] and Figurativeness×Odor [F(4,184) = 2.5; p = 0.041]. Re-referenced normalized sweetness ratings were well above zero for all Image×Odor×Figurativeness conditions, which is also reflected in the significant intercept test of the ANOVA [F(1,46) = 855; p<0.001]. This indicates that in the presence of odors and images, sweetness ratings are enhanced compared to ratings for the sucrose-alone stimuli presented at the beginning of the session. Bonferroni-corrected post-hoc comparisons revealed no significant pair-wise sweetness differences for Image but significantly higher sweetness ratings for strawberry-flavored stimuli compared to caramel-flavored stimuli (p = 0.003). Strawberry-flavored stimuli ratings were not significantly different from savoury-flavored stimuli, but showed a trend towards higher ratings for strawberry (p = 0.053).

**Table 3:** Effects of Image, Odour and Figurativeness on sweetness ratings.

Factor	F(df,df)	p
Image	3.2(2,92)	0.045
Odour	5.4 (2,92)	0.006
Figurativeness	5.5 (1,46)	0.023
Image*Figurativeness	5.0 (2,92)	0.009
Odour*Figurativeness	0.9 (2,92)	ns
Image*Odour	2.5 (4,184)	0.041
Image*Odour*Figurativeness	1.2 (4,184)	ns

### Effects of Odor and global Figurativeness context on sweetness ratings of imageless stimuli

**Figure 3:**
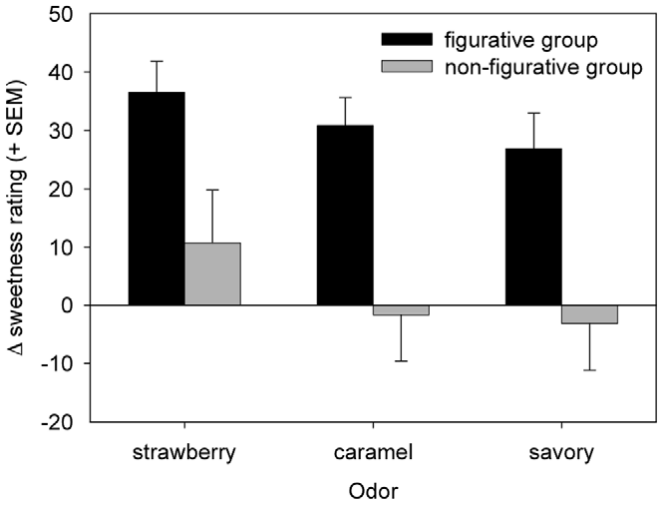
Sustained sweetening effect of Image context. Re-referenced sweetness ratings for odor-taste stimuli during no-image trials, presented in a context where either figurative or non-figurative primers have been shown in between no-image trials. Ratings were compared to no-image trials that preceded the experiment and in which participants had not been exposed to any Image. The vertical axe represents the re-referenced ratings for Odor×Figurativeness: Main odor effect indicating a sustainedsweetening effect for all odors in the figurative context, and for strawberry odor in the non-figurative context.

The repeated-measures ANOVA that evaluates the contribution of Odor and the Figurativeness of the image context to sweetness ratings for no-image trials revealed significant effects of Odor [F(2,92) = 6.3; p = 0.003] and Figurativeness of the image context [F(1,46) = 11.9; p = 0.001], but no interaction effect. Note that these trials include no images, but the trials are intermixed with trials that did include images. They were compared to ratings for the sucrose-alone stimuli presented at the beginning of the session. In this comparison of imageless stimuli, the main effect of Odor was due to higher sweetness ratings for strawberry odor stimuli compared to both the caramel odor stimuli [p = 0.010] and the savoury odor stimuli [p = 0.021], respectively (see Figure 3). The significant effect of Figurativeness of the image context was due to consistently higher sweetness ratings for imageless stimuli presented in a context of figurative images, compared to the context of non-figurative images.

### Effects of Image and image Figurativeness on sweetness ratings for all stimuli

**Figure 4:**
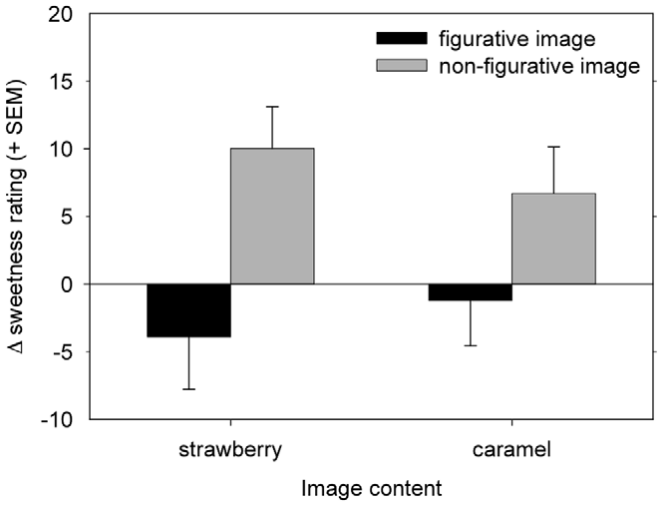
Immediate sweetening effect of Image presentation. Re-referenced sweetness ratings for odor-taste stimuli during image trials, compared to the sustained sweetening effect of an image context (see Figure 3). The vertical axe represents the *additional* sweetening effects of Image trials compared to no-image trials within an image context. Note that ratings were not compared to baseline Odor-Taste ratings which included no exposure to Images at all. Image×Figurativeness: Compared to the no-image trials within an image context, there is an increase in sweetness rating when a Non-Figurative image is present. The trials with a figurative image, however, do not show an additional increase in sweetness ratings. We cannot exclude the possibility that this is due to a ceiling effect of the figurative context (see Figure 3). The negative effect of Figurative Images was not significant.

Compared to the no-image conditions, sweetness ratings for stimuli that were visually primed with strawberry or caramel images within the trials, showed *additional* sweetness effects. These effects are reflected in the significant Figurativeness×Image interaction presented above. When examining sweetness ratings for the two Image conditions (Strawberry or Caramel) and the two Figurativeness conditions in comparison with the imageless conditions, sweetness ratings were relatively highest in the trials including non-figurative images, and differed more between figurative and non-figurative conditions in the case of strawberry images than in the case of caramel images (Figure 4).

### Effects of image-odor congruency on sweetness

**Figure 5:**
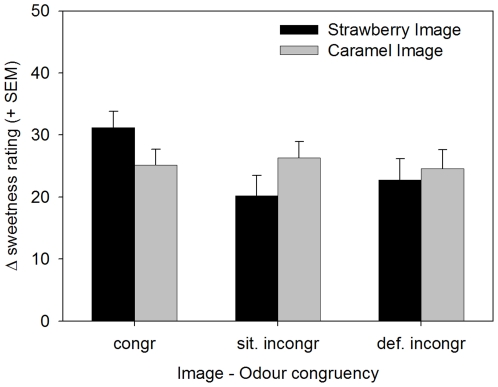
Sweetness ratings for 3 Conguency levels: Congruent, Situational Incongruent, Definitive Incongruent. The vertical axe displays the rereferenced sweetness ratings. Image×Congruency Interaction: Strawberry but not caramel image shows higher sweetness ratings for stimuli with congruent image-odor combinations compared to situational incongruent odor-image combinations.

The repeated-measures ANOVA that tested for effects of image-odor congruency on sweetness ratings revealed no main effect of Image and failed to reach significance for Congruency [F(2,92) = 2.3; p = 0.105]. However, the Image×Congruency interaction was significant [F(2,92) = 4.3; p = 0.016]. This interaction was caused by higher sweetness ratings for stimuli with congruent image-odor combinations than for situational incongruent odor-image combinations, but only if the image was strawberry (Figure 5). For caramel images, this difference was not observed and for definitively incongruent odor-image combinations (involving savoury odors) the image did not affect sweetness ratings differentially.

## Discussion

The degree of recognizability of visual information influences flavor perception differently. Taste enhancement through visual priming depends on semantics: figurative images enhanced sweetness ratings more than non-figurative images. Therefore, we suggest that sensory integration appears to include aspects of (visual) memory. The results have implications for theories and further research on multimodal sensory integration in general.

### Figurative and Non-Figurative images affect flavor differently

The priming of participants with images of sweet-tasting foods enhanced subsequent sweetness ratings. In fact, if responses to imageless stimuli are compared to responses to stimuli preceded by images, the figurative images caused a substantial enhancement for both caramel and strawberry images. Non-figurative images caused smaller, but consistent decreases of sweetness ratings for caramel and savoury odor. Only strawberry flavor was influenced by preceded non-figurative strawberry images. Note that the effect of figurativeness was found in imageless trials: the participants in the ‘figurative group’ produced higher sweetness ratings than the participants in the ‘non-figurative group’. This difference is found in spite of the fact that responses for all participants were referenced to their individual responses to 6% sucrose solutions. Subsequent evaluation of the trials including images showed an additional sweetness enhancement by non-figurative images and a selective enhancement by figurative strawberry images. The combination of odors and images produced a general sweetness enhancement compared to the 8% sucrose-only stimuli. It should be noted here that since this enhancement was also apparent in imageless stimuli and since no odorless stimuli were presented, it remains unclear whether the overall sweetness enhancement is due to the presence of odors, the image context (in neighbouring stimuli) or a combination of the two. In spite of this uncertainty, the demonstrated sweetness differences between stimuli of different odor and image categories underline the relevance of both factors for sweetness perception.

We defined two image categories with different degrees of recognizability. Figurative images are easily recognizable and may therefore elicit stronger memories of the food product. Memories of earlier taste experiences are known to influence flavor perception in a widespread and long-lasting way [29]. This is illustrated by the influence of congruency of stimuli of different sensory modalities: stronger cross-modal effects are observed for (semantically) associated colour-odor pairs compared to un-associated pairs (Gilbert, Martin, & Kemp, 1996; Zellner & Kautz, 1990). It has been suggested that after (implicit) learning of new combinations from different sensory modalities, a form of synesthesia occurs: An encounter with a stimulus of one sensory modality elicits involvement of the associated sensory modality also [30]. This may especially be true for visual images, as vision is found to dominate other senses (Schifferstein & Cleiren, 2005). Indeed, neuroimaging research shows that learned associations between images and smells result in co-activation of primary sensory cortices, in addition of the hippocampus (known for its role in memory processing). Subjects that were trained to associate a certain smell with a certain image, activate the piriform cortex (smell), when previously associated images are now shown without the smell [21]. This suggests that they are not only ‘remembering’ the odor that accompanied the image, but they are actually ‘smelling’ it. Thus, in our behavioral study, olfactory and taste areas may have been activated stronger in reaction to figurative images, than to non-figurative images. Through the mechanism of synesthesia, this may result in the perception of a sweeter product. Our results suggest that this effect may be larger for figurative visual information, compared to non-figurative visual information. Further neuroimaging research directly comparing both forms of visual priming is needed to confirm this.

So, it appears that figurative and non-figurative information affects perceived sweetness in a different way: non-figurative priming (i.e. colour and texture features) induces a transient sweetening effect that is present immediately after visual priming. However, figurative visual priming evokes a longer lasting effect that is also present during trials *without* visual priming and may be the result of memory involvement and subsequent synesthesia. In real life, it is most likely that immediate and memory-based integration occur simultaneously all the time (as evidenced by the transient and sustained components that we find), but each may be evoked differentially and to different extends by different (figurative and non-figurative) information.

**Figure 6:**
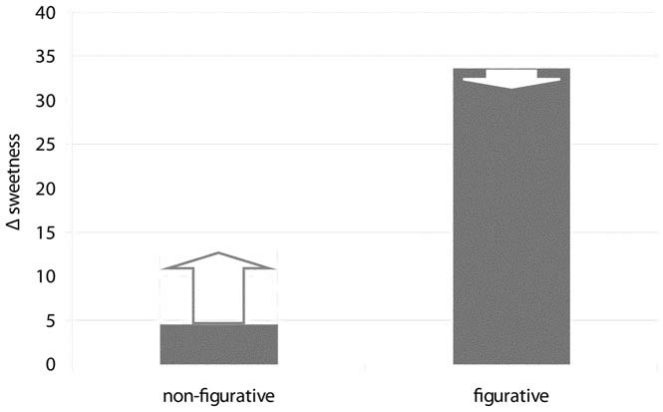
Relative magnitudes of the sustained and transient image effects: sweetening effects of the image and no-image trials combined (sum **Figures 3** and **4**). The vertical axes displays referenced sweetness ratings. Additional sweetening effect of a non-figurative image within a non-figurative context, but no additional effect of a figurative image within a figurative context. Figurative context increases sweetness ratings almost to a maximum sweetness rating leaving no room for an additional increase in sweetness rating when a figurative image is present within the trial.

See Figure 6 for an illustration of the relative magnitudes of the sustained and transient image effects.

A possible explanation for these findings may be the psychological (neural) mechanisms behind visual priming. First, it could simply reflect a *magnitude* effect. If the effect of figurative priming was stronger than non-figurative priming, it may have taken longer to decrease. However, the problem of what determines the difference in magnitude remains unresolved. Second, it may also reflect a *categorization* effect [25]: while figurative priming elicits earlier memories of actual sensory experiences with the depicted foods, non-figurative priming may only induce a general gist of the sensory qualities of the food, based on non-specific experience with foods of similar colour and texture. The non-specificity of the image will include the experience with similarly coloured but non-sweet foods. For example, red is most often associated with sweet fruits but also related to non-sweet foods such as peppers, tomatoes or raw meat. Similarly, brown colours could suggest sweet, chocolate-like foods (or caramel as in this study) but it is also associated with bread or fried foods. Indeed, 75% of our participants reported no specific (conscious) association with any kind of food product after the non-figurative visual priming. Therefore, our non-figurative images can be considered ambiguous, abstract visual information. This kind of visual information has been found to activate implicit memory processes for a longer period of time [31], possibly due to the observer's attempt to categorize the information into more explicit semantic categories [32]. Hence, participants may have used their implicit memory system to keep the image active, but its actual meaning (and thus priming effect) may have become clear only *in combination with* the actual taste-odor sensory qualities. We hypothesize that this is why the non-figurative images result in a transient effect only and not in a general and sustained priming effect that is based on explicit object recognition and subsequent immediate semantic categorization.

Summarising, based on these behavioral results, we propose that figurative priming may elicit a memory process with a sustained priming effect (synesthesia) as a result, while more ambiguous non-figurative priming does have a transient effect in combination with other sensory modalities during actual sensory integration but appears not to induce a general priming effect. Obviously, formal testing is required to confirm this suggestion.

### Different odors respond differently to visual priming

In the present study, we tested whether odors of two different sweet-tasting products (caramel and strawberry) contributed to the sweet taste of sucrose solutions. We also included a savoury odor. Of the two ‘sweet’ odors used, strawberry odor resulted in higher sweetness ratings compared to a savoury control odor. In addition, the strawberry odor also caused higher sweetness ratings than the caramel odor. These odor effects applied both for trials with- and trials without image presentation. Previous studies have demonstrated that different odors influence multisensory processing to different degrees [33]. Strawberry odor in particular was previously reported to be the most sweetening odor among others [34]. This may be caused by a difference in intensity or previous association with an odor-taste combination [35]. In the case of the present study, differences in odor intensity are an unlikely explanation for differences in sweetness enhancement since intensities of the three odors were matched. Contrastingly and not in line with expectations, the sweetness contribution of caramel odor did not exceed that of the savoury odor. Although the caramel and the savoury odors used are known references for very distinct odor qualities, the chemical components involved are structurally similar. Possibly, the initially different associations with product categories for these two odorants became diffuse due to the consistent co-presentation of the savoury odorant with sucrose solutions. This would explain the similar contributions for both odors to sweetness and their consistently lower contribution to sweetness than strawberry odor. Although the chemical complexity of the used odors is not the same for all three odors (abhexone and furaneol consist of a single odorant whereas the strawberry odor is a mixture of more than 10 different odor components) it is unlikely that the mere complexity of chemicals affected the odor-taste association. Evidence for this comes from work showing that the neural activations in the rat pyriform cortex are not defined by the odor's chemical complexity but by the circumstances under which it was experienced: If the mixture was presented as a unitary stimulus, the pyriform neural representation after repeated exposure is also unitary [18]. In humans, mixtures of odorants, each with a distinct smell, produce new but perceptually inseparable odors if the mixtures consist of as few as three or more components [36]. This poor ability to distinguish elements in a mixture was shown to be identical for the case that each composing element itself consisted of a mixture with a distinct smell [37], [38]. Hence, singular odorants and mixtures are processed in similar ways, provided that the mixtures represent a known unitary odor quality, as was the case with the strawberry odor in this study. Nevertheless, the difference in complexity may have indirectly affected the sweetness enhancing ability. Naturally occurring odors, like caramel and strawberry, are generally produced by complex mixtures of odorants. Although the single components furaneol and abhexone are important contributors to natural caramel and savoury odors, their smells may appear different to what participants are used to. If the qualitative deviation from the natural odors is large enough, it may be expected that their taste-modulating capacities will be reduced accordingly. If the more complex strawberry odor was perceived as more ‘natural’ than furaneol and abhexone, this may explain the difference in sweetness modulating capacity between these odors.

### Congruency

We hypothesised that food images would enhance sweetness perception most if they were congruent with the odors embedded in the taste stimuli. In the case of strawberry images, this hypothesis is supported by the data: Sweetness ratings were higher when strawberry images were combined with strawberry odors than when the same images were combined with caramel odors. In addition, the same strawberry odor produced lower sweetness results when combined with caramel images. Very different from this, however, were the results for sweetness ratings as a function of image-odor congruency when caramel images were involved. Possibly, this was caused by the fact that caramel and savoury odors may have been ambiguous stimuli for reasons discussed above.

### Sensory Integration

Sensory integration is usually studied using bimodal integration of color and odor on taste [10]. However, in real-life, sensory integration is more complex and usually involves more than two modalities and presumably even involves memory processes. To broaden our view on sensory integration, the present study includes a third modality and finds that the complexity of sensory integration increases with an increasing number of modalities. Also, the use of figurative and non-figurative images approaches real-life sensory integration by including memory, as another aspect of vision (texture and recognizability) in addition to the influence of previously studied colors. Even within one modality, varying the recognizability of the visual information has a different effect on the mechanism of multimodal integration processes. Therefore, we believe that our results have implications for theories about the way the brain integrates (sensory) information in general. For example, we suggest that the time frame in which a certain modality exerts its influence is important to consider. Also, Schifferstein et al [1] described the possibility of one modality dominating the influence of others. Figurative visual information may dominate other sensory modalities because it identifies the food product involved relatively easily and detailed, compared to odors and taste, probably by including memory-induced synesthesia. Most importantly, the mechanisms of trimodal or multimodal, sensory integration in general, may differ from those at work during bimodal sensory integration.

### Limitations

The sweetening effect of the figurative images was a sustained effect that remained present during no-image trials after participants were exposed to the figurative image in between trials. To our surprise, we did not find an additional sweetening effect during the image-trials for figurative images. Our data do not permit us to conclude that additional transient priming is absent during figurative visual priming, due to a possible ceiling effect. Participants were presented with 6% sucrose solutions during experimental trials. Before experimental trials, they received 4%, 6% and 8% sucrose solutions, which may have influenced their range of rating sweetness levels for all trials afterwards. After figurative visual priming, sweetness ratings often reached close to maximum levels, which may indicate a sweetness rating similar to that of an 8% sucrose solution. It is possible that if, prior to the experimental conditions, we would have presented participants with higher sucrose solutions (thereby inducing an expected range of sweetness ratings to include values that well exceed those of our experimental conditions) a transient effect for figurative priming may have been present as well.

Second, when offering people a limited number of attributes to rate the respective perceived intensities of stimuli, they may perceive differences between stimuli that cannot be expressed by the available attributes. For instance, in the present study, we asked subjects to rate sweetness intensities for stimuli that also varied in odor properties. For such cases, it has been suggested that observers will attempt to account for the perceived qualitative differences in their responses by ‘dumping’ part of the unaccounted variation in the attributes asked for. This process is referred to as ‘halo dumping’ [39] [40], which is considered a response-bias that may contribute to the observation of cross-modal interactions. Being aware of the risks that this posed for the present study, we nonetheless limited the response attribute set to ‘sweetness’ considering that we were primarily interested in the modulation of odor-taste interactions by the image context. Because halo dumping is considered a response bias introduced by the conscious awareness of stimulus aspects unaccounted for by available attributes, the low incidence of participants that reported having noticed odors (21%) suggests that the interactions observed in the data are predominantly due to cross-modal interactions rather than to halo dumping. Apart from these odor-taste interactions, we assumed that the manipulation of image context did not introduce similar ‘halo dumping’ effects when subjects are asked to evaluate taste intensities.

### Conclusion

A variation in the recognizability of visual information, (measured with images with equal visual texture qualities), reveals different effects on flavor sweetness ratings. Figurative image context exerts a longer-lasting sweetening effect on these taste-odor combinations. Non-figurative visual information exhibits a, transient effect that is only present when the visual stimulus is presented simultaneously with the taste and odor stimuli. This difference may reflect differences in visual information processing and its activation of semantic memory. The sweetening effect of visual information is independent of its congruency with the taste-odor combinations, even when the odor represents a qualitatively different taste category.

The results indicate that some aspects of multimodal (sensory) integration such as the duration of influence of a stimulus, and sensory dominance should be taken into account. They may have implications for theories on multimodal sensory integration in general. The current study involved behavioral data. Future neuroimaging research can enhance our understanding of how the brain operates during multimodal sensory integration.
